# Vogt-Koyanagi-Harada Disease: A Narrative Review

**DOI:** 10.7759/cureus.58867

**Published:** 2024-04-23

**Authors:** Ayushi Tayal, Sachin Daigavane, Nivesh Gupta

**Affiliations:** 1 Department of Ophthalmology, Jawaharlal Nehru Medical College, Datta Meghe Institute of Higher Education and Research, Wardha, IND

**Keywords:** vkh syndrome, rheumat, ophthalmology, bilateral uveitis, idiopathic uveitis, vogt-koyanagi-harada disease

## Abstract

Vogt-Koyanagi-Harada (VKH) disease is an idiopathic immune-related sickness that affects multiple systems and melanocytes in organs such as the uvea, ear, and meninges. The primary cause of activity is cellular immunological responses. Vogt-Koyanagi disease is identified primarily by skin abnormalities and anterior uveitis. Harada's illness is distinguished by neurological symptoms and exudative retinal detachments, which are associated with the *HLA-DR4* and *HLA-DRw53* genes. Pigmented races, such as Hispanics and Native Americans, are more likely to have VKH disease. Clinical features are blurred vision, floaters, alopecia, vitiligo, diffuse choroidal inflammation with disc edema, and exudative retinal detachment. Differential diagnoses include posterior scleritis, uveal effusion syndrome, central serous chorioretinopathy, and sympathetic ophthalmitis. The investigations used are optical coherence tomography (OCT), fundus fluorescein angiography (FA), and B-scan ultrasonography (USG). Treatment is done by using systemic steroids, cycloplegics, and immunosuppressants.

## Introduction and background

Vogt-Koyanagi-Harada (VKH) disease is an idiopathic immune-related disease that affects multiple systems and melanocytes in organs such as the uvea, ear, and meninges. It is primarily driven by cellular immunological responses [1]. Skin abnormalities and anterior uveitis are the primary distinguishing features of VKH. Harada disease is characterized by neurological symptoms and exudative retinal detachments. The choroid is the primary site of inflammation, with the potential involvement of the iris and ciliary body [2,3]. Although VKH syndrome rarely affects children, a three-year-old female with severe bilateral panuveitis, significant optic disc enlargement, and serous retinal detachment in both eyes was reported to have a case of a comparable illness by Katsuyama et al. [4]. Because of severe ocular problems brought on by recurring inflammation, the clinical course is often aggressive, and the visual prognosis is worse than in adult patients.

Clinical signs of VKH disease include panuveitis, cerebral fluid pleocytosis, skin conditions such as loss of skin color in patches, baldness, and anomalies of the inner ear. The most common ocular changes observed in the initial stages of VKH disease are serous retinal detachment, iridocyclitis, and optic disc edema. Studies have shown that VKH disease involves the choroid and is brought on by an autoimmune response against melanocytes, which express the tyrosinase family of proteins [5-9]. Previous investigations using pathological specimens have clarified the role of melanocytes and other cells in VKH illness. Hyperreflective foci and other chorioretinal cellular components may be distinguished by optical coherence tomography (OCT), according to prior research [10-12]. Apart from healthy eyes, hyperreflective foci have been observed in the choroid or retina of eyes suffering from a range of conditions such as choroideremia [12], age-related macular degeneration [13], and diabetic retinopathy [14]. There have been reports linking them to both the activity and progression of the disease [15]. According to current research, melanocyte pigmentation in the normal choroid is the source of hyperreflective choroidal foci (HCF), which are quantifiably measured by enface OCT [16,17]. Sunset glow fundus (SGF), one of the hallmarks of VKH sickness, may be connected to alterations in choroidal components such as melanin pigment [18,19]. It is still unclear exactly how HCF and fundus pigmentation levels relate to one another quantitatively. In light of this, we postulated in this work that HCFs on enface OCT images in patients with VKH disease would differ from those of normal people and vary with fundus color. In eyes with normal fundus and VKH illness, we looked into the relationship between the degree of fundus pigmentation and HCF on enface OCT pictures. The variations in HCF distribution between the eyes of normal controls and those affected by VKH illness with and without sunset glow were also compared.

Clinically, VKH disease is divided into two phases: acute with first onset and chronic with repeated episodes [20]. The intensive use of combined steroidal and nonsteroidal immunosuppression for extended periods of time, given right at the start of treatment, is the usual regimen for initial-onset VKH illness [21]. It has been shown that in a small percentage of VKH patients, delayed tapering of systemic corticosteroid therapy leads to exacerbation since nonsteroidal immunosuppression is not linked to this kind of treatment, even when severe immunosuppressive therapy is started from the start. On the other hand, rapid tapering is necessary when corticosteroid treatment is interrupted by the onset of adverse effects, such as gastrointestinal ulcers, diabetes, osteoporosis, and, less frequently, corticosteroid-induced psychosis. The illness advances to the chronic recurrent stage if adequate levels of inflammatory control and/or clinical quiescence are not reached [22-24]. The clinical indicators that are present in the eyes include pigment migration, peripheral atrophic foci that are scars from Dalen-Fuchs nodules, chronic granulomatous anterior uveitis, and sunset glow fundus (SGF), which is described as progressive fundus depigmentation [25].

This paper reviews the disease's clinical characteristics, results, complications, and recent treatment approaches. It also highlights new findings in the field's understanding of the immunogenetics and pathophysiology of VKH disease.

## Review

Epidemiology and pathogenesis

Pigmented races, such as Hispanics and Native Americans, are more likely to develop VKH disease. The disease strikes females more commonly than males. Of 65 participants with VKH disease who participated in one research study, one was Asian Indian, one was Native American, and the remaining demographics were as follows: 78% were Hispanic, 10% were Asian, 6% were Black, and 3% were Caucasian [26]. Of this study's participants, 74% were female. The average age of those who fell ill was 32 years old, and most were in their second or fifth decade of life. Certain HLA genotypes (DRB1 * 0405) are associated with an elevated risk. It suggests a genetic predisposition to VKH illness [27,28]. The underlying etiology of the condition is unknown.

Clinical features

The sickness is divided into four clinical phases: prodromal, uveitic, chronic, and recurrent [29].

Prodromal Phase

Patients may experience flu-like symptoms such as nausea, fever, and headaches. Within 1-2 days of commencing the prodromal phase, the patient reports blurred vision, photophobia, conjunctival redness, and ocular pain. During the prodromal phase, some notice touch sensitivity in their scalp, hair, and skin.

Uveitic Phase

During the initial stage of uveitis, a few cells may be present in either the anterior chamber (AC), the vitreous, or both. The posterior choroid thickens. The optic disc becomes edematous and hyperemic. Several serous retinal detachments or small folds protrude from the macula. In severe circumstances, separation might become bullous.

Chronic Phase

The elevation of the neural retina gradually decreases after systemic corticosteroid treatment as the fluid in the subretinal space is absorbed. The anterior chamber and vitreous cells reduce. After a few months, the fundus may become depigmented, resembling a sunset. Small, discrete, and dispersed depigmented lesions can be found inside the sunset glow fundus. The most common cause of these abnormalities is retinal pigment epithelial degeneration or loss [8].

Depigmentation in the corneal limbus is also occasionally seen after a month of onset. This limbal depigmentation is called Sugiura's sign.

Recurrent Phase

Compared to posterior uveitis, anterior uveitis is more common in recurrent instances. Signs of chronic iridocyclitis such as Koeppe iris nodules, posterior synechiae, and mutton-fat keratic precipitates are all signs of the recurrent stage. The iris loses some of its color and appears atrophic. Choroidal neovascularization (CV) can occur in the macula and peripapillary region, frequently causing hemorrhage. Retinal pigment epithelium (RPE) proliferation may result in subretinal fibrosis.

Systemic Associations

Neurological symptoms may include headaches, fever, nausea, vomiting, and neck stiffness. Auditory signs include vertigo, tinnitus, and sensorineural hearing loss (which are often present at the start of the disease). Vitiligo may emerge on the hands, face, lower back, and shoulders 2-3 months post-VKH commencement. Other symptoms include poliosis and baldness.

At the First International Workshop on Vogt-Koyanagi-Harada sickness, the consensus committee discussed and reported on the updated diagnostic criteria for VKH sickness. The diagnostic criteria for Vogt-Koyanagi-Harada disorders are as follows: no history of penetrating ocular trauma or surgery preceding the initial onset of uveitis and no clinical or laboratory evidence suggestive of other disease entities.

Early manifestations of the disease include evidence of diffuse choroiditis (with or without anterior uveitis, vitreous inflammatory reaction, or optic disc hyperemia), which may manifest as focal areas of subretinal fluid or bullous serous retinal detachment. With equivocal fundus findings, the following must be present as well: focal areas of delay in choroidal perfusion, multifocal areas of pinpoint leakage, large placoid areas of hyperfluorescence, pooling within the subretinal fluid, and optic nerve staining by fluorescein angiography (FA). Clinical features also include diffuse choroidal thickening, without evidence of posterior scleritis by ultrasonography (USG).

Late manifestations of the disease include a history suggestive of prior presence of findings, ocular depigmentation, sunset glow fundus, Sugiura's sign, and other ocular signs such as nummular chorioretinal depigmentation scars, retinal pigment epithelium clumping and/or migration, and recurrent or chronic anterior uveitis; neurological/auditory findings such as meningismus (malaise, fever, headache, nausea, abdominal pain, stiffness of the neck or back, or a combination of these factors), tinnitus, cerebrospinal fluid (CSF) pleocytosis; and integumentary findings such as alopecia, poliosis, and vitiligo.

**Figure 1:**
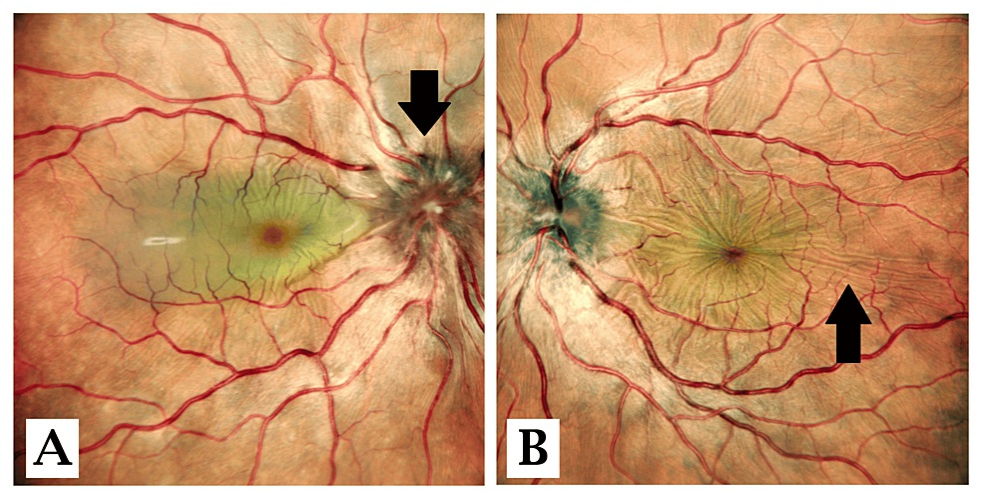
Fundus image of the right eye (A) and the left eye (B) A: The fundus of the right eye with severe disc edema (black arrow) and few choroidal folds. B: The fundus of the left eye with disc edema and multiple choroidal folds (black arrow). Image credit: Dr. Nivesh Gupta

Figure 1A shows the fundus of the right eye having severe disc edema (black arrow) and few choroidal folds. Figure 1B shows the fundus of the left eye having disc edema and multiple choroidal folds (black arrow).

Ancillary investigations

Fluorescein Angiography (FA)

**Figure 2:**
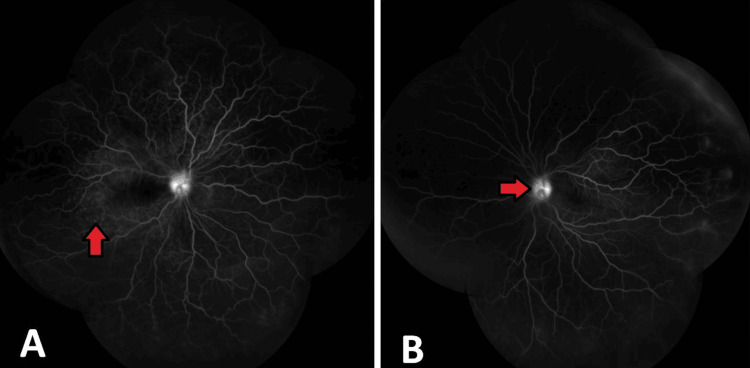
FA montage image of the right eye (A) and the left eye (B) A and B: FA montage images showing multiple pinpoint leakages along with hot discs present in the right and left eye (red arrows). FA: fluorescein angiography Image credit: Dr. Nivesh Gupta

In the early stages of VKH disease, FA reveals many hyperfluorescent lesions at the RPE level. These areas tend to grow gradually. The dye collects in the subretinal space after seeping through the RPE. In the chronic phase, angiography reveals diffusely scattered hyperfluorescent lesions caused by RPE window defect abnormalities. Approximately 70% of individuals may exhibit disc leakage while they are in the acute stage of the illness [30]. Figure 2A and Figure 2B (red arrow) show FA montage images showing multiple pinpoint leakages along with hot discs present in the right and left eye, respectively.

Areas of linear hypofluorescence can be seen in some cases, which are usually caused by the presence of choroidal folds. When compared to birdshot chorioretinopathy, retinal vascular leakage and sheathing are uncommon. A useful metric for tracking the effectiveness of the first corticosteroid treatment is the presence and size of pinpoints [31,32].

Indocyanine Green Angiography (ICGA)

In the early and mid phases, ICG exhibits early choroidal stromal vascular hyperfluorescence as well as hypofluorescent black patches mostly found posteriorly. During the active stage, hypofluorescent lesions are converted into hyperfluorescent lesions. Hypofluorescent dark patches can be seen during all ICG phases in the chronic stage. It might additionally help us to understand the causes and consequences of chorioretinal inflammation-related illnesses [33].

Optical Coherence Tomography (OCT)

**Figure 3:**
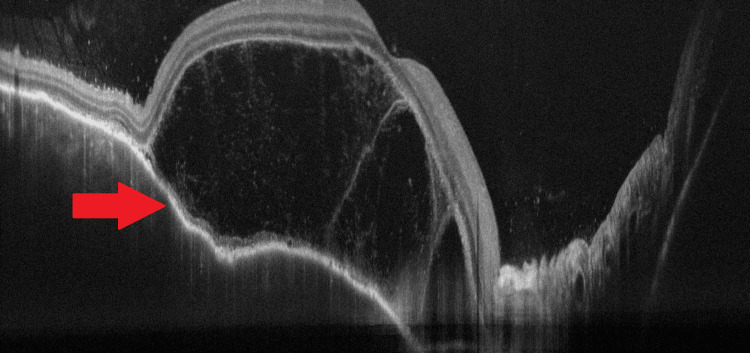
OCT image OCT image having an altered foveal contour with a large subretinal fluid pocket present with vitreous cells present (red arrow). OCT: optical coherence tomography Image credit: Dr. Nivesh Gupta

It indicates the existence of subretinal fluid. There are several septa that form fluid pockets in the outer retina. In the presence of choroidal folds, OCT indicates corrugation of the retinal epithelium/choroid as well as choroidal thickness. Increased choroidal thickness can be seen in enhanced depth imaging (EDI) OCT. Figure 3 (red arrow) shows an OCT image having an altered foveal contour with a large subretinal fluid pocket present with vitreous cells present.

These anomalous features were all found in the outer photoreceptor layer or beneath the line that represents the external limiting membrane. Some authors propose that fibrin and the damaged outer segment of the rods and cones are the sources of these strands [34-36].

Patients with the "sunset glow" fundus and chronic VKH disease were studied by Vasconcelos-Santos et al. [37]. Spectral domain OCT demonstrated thickening of the Bruch's membrane/RPE in regions where pigmented scars are present and weakening of the RPE/Bruch's membrane in atrophy areas, while "sunset glow fundus" regions have normal retinal architecture [38].

Ultrasonography

In the acute stage, ultrasonography shows diffuse thickening of the choroid, opacities in the vitreous, serous retinal detachments, and scleral thickening.

Given that it allows for differentiation between choroidal involvement in leukemia or lymphoma, diffuse malignant melanoma, and posterior scleritis, USG may be a crucial diagnostic tool [39].

Electroretinography

During the long-term phase, full-field electroretinography (ERG) exhibits diffusely decreased amplitudes in both the photopic and scotopic phases.

An evaluation of the extent of functional impairment resulting from inflammatory damage to retinal components can be made with an electroretinogram (ERG) [40,41].

Laboratory Investigation

In the early stages, cerebrospinal fluid examination reveals pleocytosis (which can last up to eight weeks) and elevated protein levels.

Treatment

VKH disease is treated with systemic steroids in high doses and then tapered slowly. Immunosuppressive medication is administered to prevent recurrences and in patients who are unable to consume steroids. High-dose corticosteroids are administered intravenously or orally during the disease's early stages. Steroids are gradually decreased over the course of six months. Lai et al. [42] reported that patients who had treatment for less than six months had a higher recurrence rate (58.8%) than those who received treatment for six months or more (11.1%). Azathioprine, methotrexate, cyclosporine, and mycophenolate mofetil are among the most effective immunosuppressants. It is crucial to remember that immunosuppressant medication should be frequently checked and examined for any adverse effects. Topical corticosteroids and cycloplegics are used until the AC cells disappear. In recurrence cases, a combination of immunosuppressive medications or biologicals is used.

Course and outcome

VKH disease-related uveitis necessitates a longer course of treatment and frequent follow-up. However, a posterior subcapsular cataract could be a side effect of steroids and a prolonged case of uveitis. Other problems include subretinal neovascularization, fibrosis, and secondary angle-closure glaucoma.

Differential diagnosis

Sympathetic Ophthalmia

It causes panuveitis and exudative retinal detachment. However, there is typically a history of ocular injury in the exciting eye. It may happen at any age, and there is no gender predisposition.

Central Serous Chorioretinopathy (CSCR)

It is often unilateral and more common in males with modest sight loss. Exudative retinal detachment can occur with multifocal CSCR. There are no vitreous or anterior chamber cells in CSCR. Separating these two illnesses is also crucial since corticosteroids, which are the first and main treatment for VKH, may increase the chance of developing CSCR [26]. Fluorescein angiography shows no disc hyperfluorescence; however, there are inkblot or smoke stack leaks. OCT reveals folds of RE, internal limiting membrane (ILM) variations, and subretinal septa in VKH, while pigment epithelial detachments (PEDs) are more common in CSCR. Stress is the primary risk factor for SC. The use of steroids can cause CSCR. The majority of instances of central serous chorioretinopathy resolve on their own within one or two months.

Uveal Effusion Syndrome

It is frequent in nanophthalmic and hypermetropic eyes with a thicker sclera. It is not typically linked with intraocular inflammation and responds poorly to systemic steroids.

Unilateral Posterior Scleritis

It shows T sign on ultrasonography (low reflectivity of fluid in the Tenon space surrounding the nerve and high reflectivity of thickened sclera). It is not connected with neurological or dermatological symptoms. Twice as many females as males suffer from posterior scleritis, a rare kind of scleral inflammation. Systemic disorders such as autoimmune diseases, lymphoma, and systemic vasculitis may be responsible for 30%-45% of cases. Posterior scleritis can also cause redness and strong ocular pain that radiates to the ears, jaw, and head, optic disc edema, serous retinal detachment, and choroidal fold and choroidal detachment. Still, it is often isolated and does not present with dermatological or neurological signs. A further hallmark of posterior scleritis is the "T sign" on ultrasonography, which is the squaring of the interface between the sclera and the optic nerve, indicating the presence of fluid in the sub-Tenon's space. Retrobulbar edema and thickening of the posterior scleral shell may also be seen [43-45].

Acute Posterior Multifocal Placoid Pigment Epitheliopathy (APMPPE)

In young and previously healthy patients, acute posterior multifocal placoid pigment epitheliopathy is an unusual inflammatory ophthalmic disorder affecting the RPE, outer retina, and choriocapillaris. Painless and rapid loss of central visual acuity (VA), either unilaterally or bilaterally, is observed after a viral prodrome. Numerous creamy white lesions may develop into chorioretinal scarring. The vitreous usually exhibits negligible or no reaction. FA typically shows late hyperfluorescence after early hypofluorescence brought on by occlusion at the site of lesions. FA outcomes set VKH disease apart from APMPPE. Hypofluorescent lesions in the middle and late stages of the disease demonstrate that ICGA enables the monitoring of the complete length of choroidal involvement. Serious retinal detachments can result from either condition and are treated with corticosteroid medication [46-48]. A wide spectrum of central nervous system (CNS) involvement, from mild symptoms (such as headache) to severe diffuse cerebral vasculitis, is described in numerous studies. In general, the prognosis for APMPPE is favorable, with rapid and/or spontaneous recovery of visual acuity. For some, visual restoration may only be partial. Systemic treatment can be given if there is a substantial visual impairment or central nervous system dysfunction [49,50].

Birdshot Chorioretinopathy

After the fourth decade of life, persistent, intraocular bilateral inflammation occurs more commonly in Caucasians. The most prevalent symptoms include disc edema, vasculitis, cystoid macular edema (CME), mild yellowish-white lesions all over the posterior pole, and chronic vitritis. The anterior segment is barely irritated. It is a chronic illness that causes progressive loss of visual acuity along with periods of exacerbation and remission. Although the outcomes of FA are less evident, ICGA typically shows hypofluorescence that persists until the later phases. The ERG results point to a possible impairment in cone and rod function. There was a significant correlation found with HLA-A29, suggesting that autoimmune mechanisms play a role in the etiology [51].

## Conclusions

VKH disease is an autoimmune panuveitis that presents with neurological, integumentary, and auditory symptoms. Although the disease's clinical characteristics have been thoroughly documented, much more has to be discovered about the disease's etiology, including hereditary and environmental triggers. Better visual outcomes and fewer ocular and systemic problems are linked to early and vigorous treatment of VKH. Furthermore, more investigation is required to comprehend the immunological dysregulation that underlies this illness.
